# Second-line anti-retroviral treatment failure and its predictors among patients with HIV in Ethiopia: A systematic review and meta-analysis

**DOI:** 10.1371/journal.pgph.0003138

**Published:** 2024-04-23

**Authors:** Gizachew Ambaw Kassie, Getahun Dendir Wolda, Beshada Zerfu Woldegeorgis, Amanuel Yosef Gebrekidan, Kirubel Eshetu Haile, Mengistu Meskele, Yordanos Sisay Asgedom

**Affiliations:** 1 Department of Epidemiology and Biostatistics, School of Public Health, College of Medicine and Health Sciences, Wolaita Sodo University, Wolaita Sodo, Ethiopia; 2 School of Anesthesia, College of Health Science and Medicine, Wolaita Sodo University, Wolaita Sodo, Ethiopia; 3 School of Medicine, College of Health Science and Medicine, Wolaita Sodo University, Wolaita Sodo, Ethiopia; 4 School of Public Health, College of Health Science and Medicine, Wolaita Sodo University, Wolaita Sodo, Ethiopia; 5 School of Nursing, College of Health Science and Medicine, Wolaita Sodo University, Wolaita Sodo, Ethiopia; Universidad de Chile, CHILE

## Abstract

Antiretroviral therapy (ART) treatment failure remains a major public health concern, with multidimensional consequences, including an increased risk of drug resistance, compromised quality of life, and high healthcare costs. However, little is known about the outcomes of second-line ART in Ethiopia. Therefore, this systematic review and meta-analysis aimed to determine the incidence and determinants of second-line ART treatment failure. Articles published in PubMed, Google Scholar, Science Direct, and Scopus databases were systematically searched. All observational studies on the incidence and predictors of treatment failure among patients with HIV on second-line ART were included. A random-effects model was used to estimate the pooled incidence, and subgroup analysis was performed to identify the possible sources of heterogeneity. Publication bias was checked using forest plot, Begg’s test, and Egger’s test. The pooled odds ratio was also computed for associated factors. Seven studies with 3,962 study participants were included in this study. The pooled incidence of second-line antiretroviral treatment failure was 5.98 (95% CI: 4.32, 7.63) per 100 person-years of observation. Being in the advanced WHO clinical stage at switch (AHR = 2.98, 95% CI: 2.11, 4.25), having a CD4 count <100 cells/mm3 (AHR = 2.14, 95% CI: 1.57, 2.91), poor drug adherence (AHR = 1.78, 95% CI: 1.4, 2.25), and tuberculosis co-infection (AHR = 2.93, 95% CI: 1.93, 4.34) were risk factors for treatment failure. In conclusion, this study revealed that that out of 100 person-years of follow-up, an estimated six patients with HIV who were on second-line antiretroviral therapy experienced treatment failure. The risk of treatment failure was higher in patients who were in an advanced WHO clinical stage, CD4 count <100 cells/mm3, and presence tuberculosis co-infection. Therefore, addressing predictors reduces the risk of treatment failure and maximizes the duration of stay in second-line regimens.

## Introduction

Anti-Retroviral Therapy (ART) is a critical turning point in the history of HIV infection, which gradually causes HIV infection to become a chronic non-fatal condition [1]. According to the Joint United Nations Programmed on HIV/AIDS (UNAIDS), approximately 38 million people worldwide were living with HIV, and 25.4 million people were receiving antiretroviral therapy (ART). Sub Saharan Africa, which disproportionately carries 70% of the global HIV burden [2, 3]. In Ethiopia, the national HIV prevalence is 1.16%. The prevalence among the urban population is 3.8% and varies from region to region, ranging from 0.54% in southern Ethiopia to 5% in Addis Ababa [4].

Antiretroviral therapy (ART) has considerably improved the prognosis of people with human immunodeficiency virus (HIV) infection [5]. Despite the remarkable progress made in HIV treatment, a considerable proportion of patients experience treatment failure, and many require second-line ART therapy. Second-line ART treatment failure is associated with multiple factors including poor adherence to medication, drug resistance, and inadequate healthcare access and provision [6].

Treatment failure is characterized by a decrease in CD4+ cell count and an increase in viral load. Second-line ART is often used as a salvage option when the first-line regimen fails [7]. Recent estimates suggest that up to 20% of people receiving ART may require second-line therapy, and this figure can reach up to 50% in some high-income countries [8, 9]. In Ethiopia, 1.5% of patients on ART use a second-line regimen [10]. It is estimated that 15% of people living with HIV experience second-line treatment failure in Sub-Saharan Africa (SSA) [9], and according to a recent study in Ethiopia, the incidence of treatment failure was 98.6/1000 person-years [11]. Previous studies indicated that patients’ CD4 cell count at the time of switching, WHO clinical stage at switch, their functional status, whether they were co-infected with TB, and their adherence to ART were all significant predictors of treatment failure [11–13].

Poor second-line ART treatment outcomes can have multidimensional consequences, including an increased risk of drug resistance, which can make future treatments more challenging [14]. It can also lead to a decline in overall health and well-being, which can negatively impact the quality of life of individuals living with HIV and increase the need for expensive third-line treatment options. Moreover, it can increase the risk of HIV transmission because individuals with treatment failure are more likely to have higher viral loads and be more infectious. When a virus is exposed to antiviral medication, its genetic makeup can change, leading to the development of drug-resistant strains. These strains can then be transmitted to other individuals, resulting in widespread outbreaks that are difficult to manage. In addition, the use of ineffective treatments can lead to more severe illness, longer hospital stays, and higher mortality rates, making it critical to identify effective treatment options and promote their widespread use to prevent the transmission of resistant viruses [14–16].

Ethiopia is one of the countries with a high burden of HIV/AIDS and has made significant progress in scaling up ART services. However, despite these efforts, some patients experience treatment failure, which can lead to the development of drug-resistant HIV strains. In Ethiopia, unlike other countries, a significant proportion of people living with HIV/AIDS reside in rural areas where health infrastructure and access to care may be limited, posing a greater challenge in managing ART failure. Moreover, there are local determinants in Ethiopia that may affect the prevalence and management of HIV/AIDS and ART failure. For instance, HIV-associated stigma and discrimination, low health literacy, and poor treatment adherence are common issues that affect the retention of patients in care [17, 18]. Furthermore, there is a dearth of systematic reviews and meta-analyses that summarize the available evidence on the prevalence of second-line ART failure and associated factors in Ethiopia. Therefore, this study aimed to determine the pooled incidence and determinants of second-line ART treatment failure in Ethiopia, by synthesizing data from previous studies. The results of this study will provide essential information for improving ART programs in other similar settings. In addition, it will inform stakeholders about the current status of second-line ART users to maximize the duration of stay on the second-line regimen and assist in planning for possible future needs.

## Methods and materials

### Study protocol registration and reporting

A systematic review and meta-analysis were performed to estimate the pooled incidence of second-line ART treatment failure among patients with HIV on second-line ART. The Preferred Reporting Items for Systematic Reviews and Meta-Analyses (PRISMA) guidelines were followed [19] (S1 Checklist). This systematic review and meta-analysis protocol was registered in PROSPERO under the reference number (CRD42024483963).

### Data sources and searching strategy

Exhaustive literature searches in PubMed, Google Scholar, Science Direct, Scopus, and online university research repositories were performed. All studies conducted on the incidence of treatment failure among patients with HIV on second-line antiretroviral therapy in Ethiopia were retrieved. The primary searching keywords included "magnitude" or "prevalence" or “proportion” or ‘burden” or "incidence" and “second line ART” and “treatment failure” or “virological failure” or “clinical failure” or “immunological failure” or “treatment outcome”; and “predictors” or “determents” or “associated factors” and “patients with HIV” and “Ethiopia”. Gray literature from the online library repository and a manual search of references were also searched. The search included all the articles published until September 2023.

### Eligibility criteria

#### Inclusion criteria

*Population*. A study was carried out on patients with HIV on second line antiretroviral therapy.

*Study setting*. Studies conducted across all regions of Ethiopia were considered.

*Study design and language*. All observational studies published in English in Ethiopia that reported the incidence and/or predictors of second line antiretroviral therapy failure.

#### Exclusion criteria

This study excluded the studies that didn’t provide information on the primary outcome of interest; and studies published as reviews, meta-analysis, and case series.

#### Outcome measurements

The primary outcome of this review was the incidence of second-line antiretroviral treatment failure, defined as clinical, immunological, virological, or a combination of those outcomes [7]. This study also examined the association between treatment failure and associated factors and expressed the log odds ratio (OR). The determinants considered in this review included ART drug adherence, advanced WHO stage, CD4 count, and tuberculosis co-infection.

### Data extraction

All articles retrieved from all databases were imported into Endnote X7, and duplicate articles were removed. Two independent reviewers, GAK and GDW, screened all articles for eligibility by first reviewing the titles and abstracts, followed by the full text. In case of any disagreement regarding study selection, a third investigator, YSA, was invited to resolve the issue. Data were then extracted from the included articles using Excel spreadsheet software. The data extraction format included author names, years of publication, regions, study design, sample sizes, number of cases, person-years of observation and quality rating, and factors (ART drug adherence, WHO clinical stage at switch, CD4 count at switch, and opportunistic infection).

### Quality assessments

The Joanna Briggs Institute (JBI) tool, adapted for observational studies, was used to assess the quality of each study [17]. The evaluation criteria included in the tool were as follows: appropriateness of the source population list; proper recruitment of study participants; sample size sufficiency; appropriateness of the study area and subject description; data analysis with sufficient coverage of the sampled data; measurement of the condition using a standard, reliable, and consistent approach for all participants; suitability of statistical analysis; adequate response rate; or use of an appropriate handling mechanism for low response rate. Each study was critically evaluated by two independent reviewers and any disagreements were resolved through discussion. If the reviewers did not agree, a third reviewer was involved in resolving disagreements between the independent reviewers. Studies were included in the final systematic review and meta-analysis if they received a total score of ≥50% on the quality evaluation checklist criteria (S2 Checklist).

### Risk of bias assessment

The included studies were assessed for bias risk by two authors, AYG and BZW, using the bias assessment tool developed by Hoy et al [20]. This tool includes ten items that evaluate bias as well as internal validity and external validity of the study. If there was any disagreement during the risk of bias assessment, the third author (MM) led a conversation to settle the issue. External validity, non-response bias, and selection bias were assessed by the first four items. The remaining six items evaluated internal validity, bias linked to analysis, and assessment bias. Research with a ’yes’ response to at least eight out of ten questions was categorized as having a ’low risk of bias.’ Studies that received a ’yes’ for 6–7 of the ten questions were classified as having ’moderate risk’ of bias, while studies that received a ’yes’ for ≤5 of the ten questions were classified as having ’high risk’ of bias (S1 Table).

### Statistical analysis

After all relevant data were extracted; they were exported from Microsoft Excel to STATA 14.1. Heterogeneity across the studies was assessed using the inverse variance (I^2^) test. I-squared value represented moderate (30–60%), substantial (60–90%), and considerable (90–100%) heterogeneity. The pooled incidence of second-line ART failure was estimated based on the Freeman–Tukey double arcsine transformation method with the Der-Simonian and Laird random-effects model because of the high degree of heterogeneity (I2 >90.1%, p <0.001). Egger’s and Begg’s tests were performed to examine publication bias.

The second outcome of this study was the factors associated with second-line ART treatment failure among patients with HIV infection in Ethiopia. For determinants of second-line ART treatment failure, the input variable required by “metan” contained the cells of the 2×2 tables; that is, the number of patients with HIV who did and who did not develop second-line ART treatment failure among exposed, and the number of patients with HIV who did and who did not develop second-line ART treatment failure among non-exposed groups in each study. All potential determinants associated with second-line ART treatment failure was determined using the hazard ratio (HR) and were calculated based on the binary outcomes of the included primary studies. The estimated pooled HR with 95% CI is reported. Finally, findings were presented in the form of forest plots with the corresponding effect size and 95% CIs.

### Ethics statement

This study did not require ethical approval.

## Results

### Study selection

**Fig 1:**
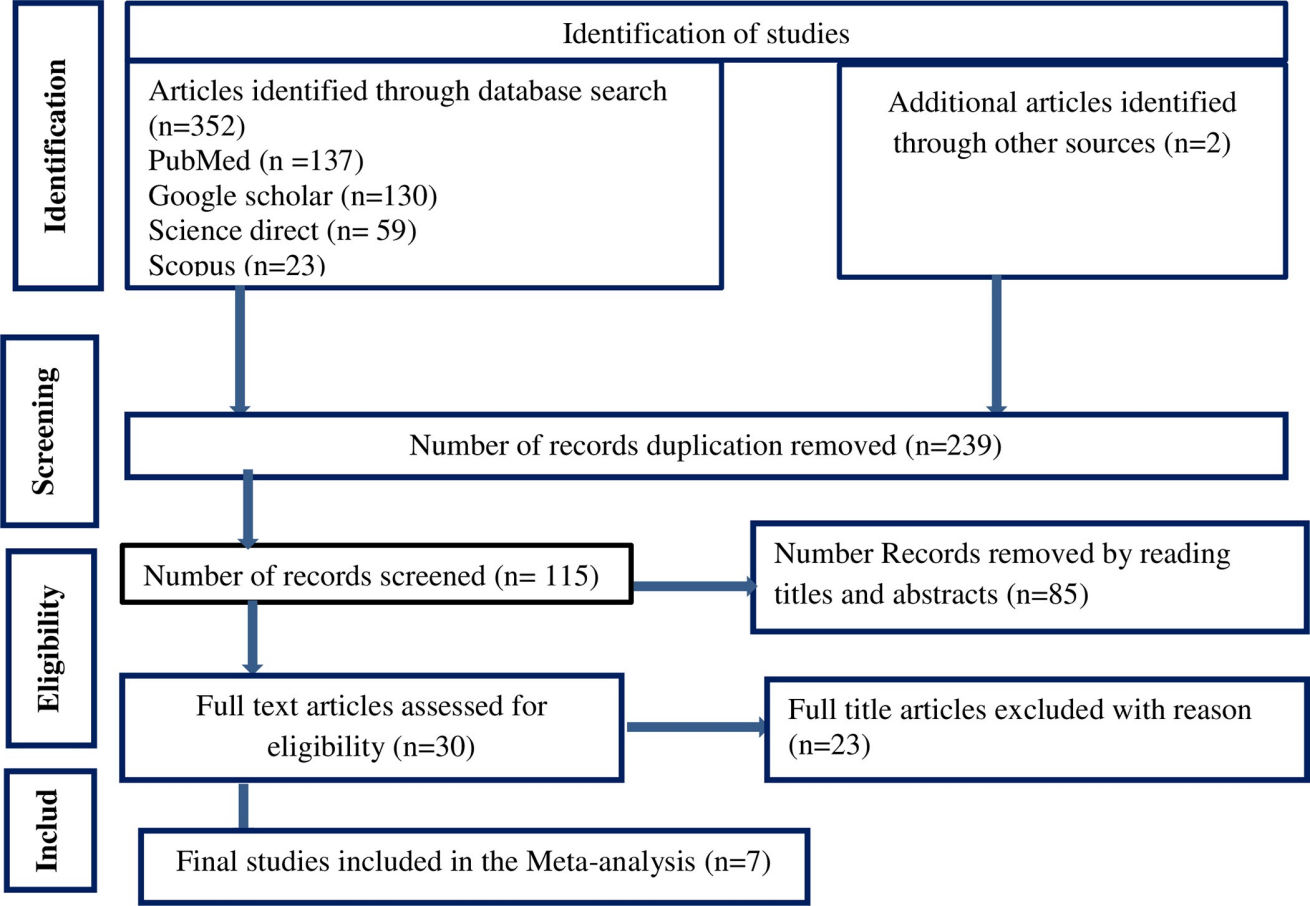
PRISMA flow diagram of articles screened and the selection process on the pooled incidence of treatment failure among patients with HIV on second-line ART in Ethiopia, 2023.

A total of 354 articles regarding second-line ART failure among patients with HIV in Ethiopia were retrieved. Of the identified studies, 239 were excluded because of duplications and irrelevancies and 85 were removed after abstract and title screening. However, 30 studies met the eligibility criteria, and 23 full-title articles were excluded for the following reasons (studies conducted outside Ethiopia, studies with insufficient data, study population not on patients with HIV, and studies that did not report the outcome interest). Finally, seven studies were included (Fig 1).

### Characteristics of included studies

A total seven studies were included in this systematic review and meta-analysis [11–13, 21–24]. This meta-analysis included 3,962 PLWHIV on second-line antiretroviral with 9,497 person years of follow-up. All studies were institution-based retrospective follow-up studies. The publication period spans from 2016 to 2023. The minimum and maximum sample sizes in Amhara and Tigray were 192 and 1, 011, respectively [11, 21]. Of the included studies, three were from Amhara [11–13], three from Tigray [21, 23, 24], and one from Addis Ababa [22] (Table 1).

**Table 1:** Descriptive summary of studies included in the meta-analysis of second-line antiretroviral treatment failure and its predictors among patients with HIV in Ethiopia, 2023.

Authors(Year)	Region	Study participant	Study design	Sample size	Number of cases	Person-years of observation	Quality rating
Alene et al (2019) [11]	Amhara	Adults	RFU	1011	254	2576	7
Giday et al (2023) [21]	Tigray	Adults	RFU	192	47	872	6
Haftu et al (2020) [23]	Tigray	Adults	RFU	227	57	789	6
Masresha et al (2021) [24]	Tigray	Adults	RFU	474	73	1480	7
Tsegaye et al (2016) [13]	Amhara	Adults	RFU	356	67	1085	6
Wodajo et al (2022) [12]	Amhara	Adults	RFU	642	39	968	7
Zakaria et al (2022) [22]	Addis Ababa	Adults	RFU	360	44	1027	7

### Pooled incidence of second-line antiretroviral therapy failure (Meta-analysis)

**Fig 2:**
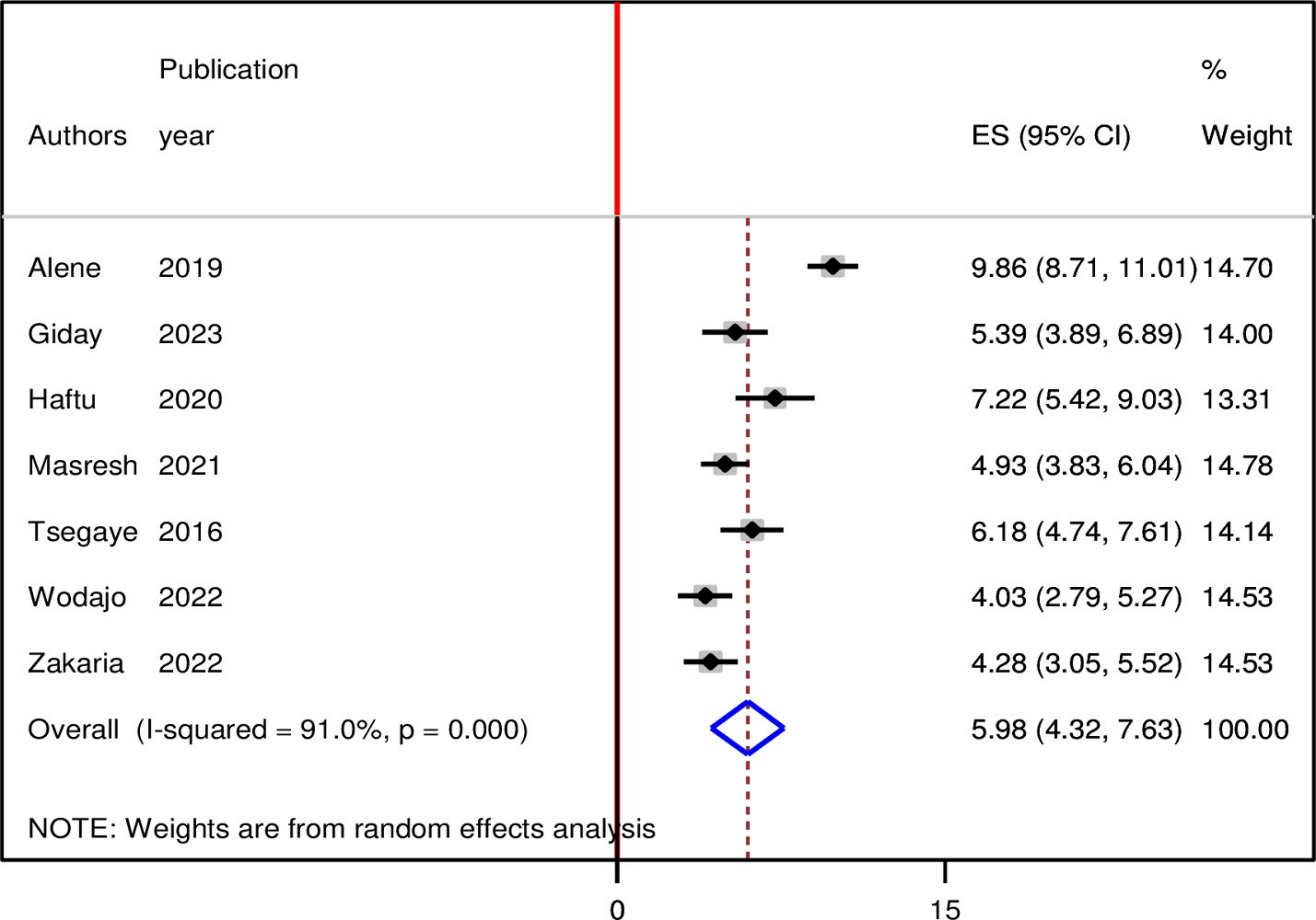
Forest plot displaying on the pooled incidence of treatment failure among patients with HIV on second-line antiretroviral therapy in Ethiopia, 2023.

Seven studies involving 3,962 patients with HIV on second-line ART reported the incidence of second-line ART failure. A total years of follow-up for the study participants was 9,497 person-years of observation In the random effect pooled incidence of second-line ART failure in Ethiopia was 5.98 (CI: 4.32, 7.63) per 100 person years of observation, with significant heterogeneity observed across the studies (I^2^ = 90.1%, p value < 0.001) (Fig 2). In terms of individual studies, the highest 9.86 per 100 years of observation and the lowest (4.03%) incidence of second-line ART failure was reported in studies in Amhara regions.

### Subgroup analysis

**Fig 3:**
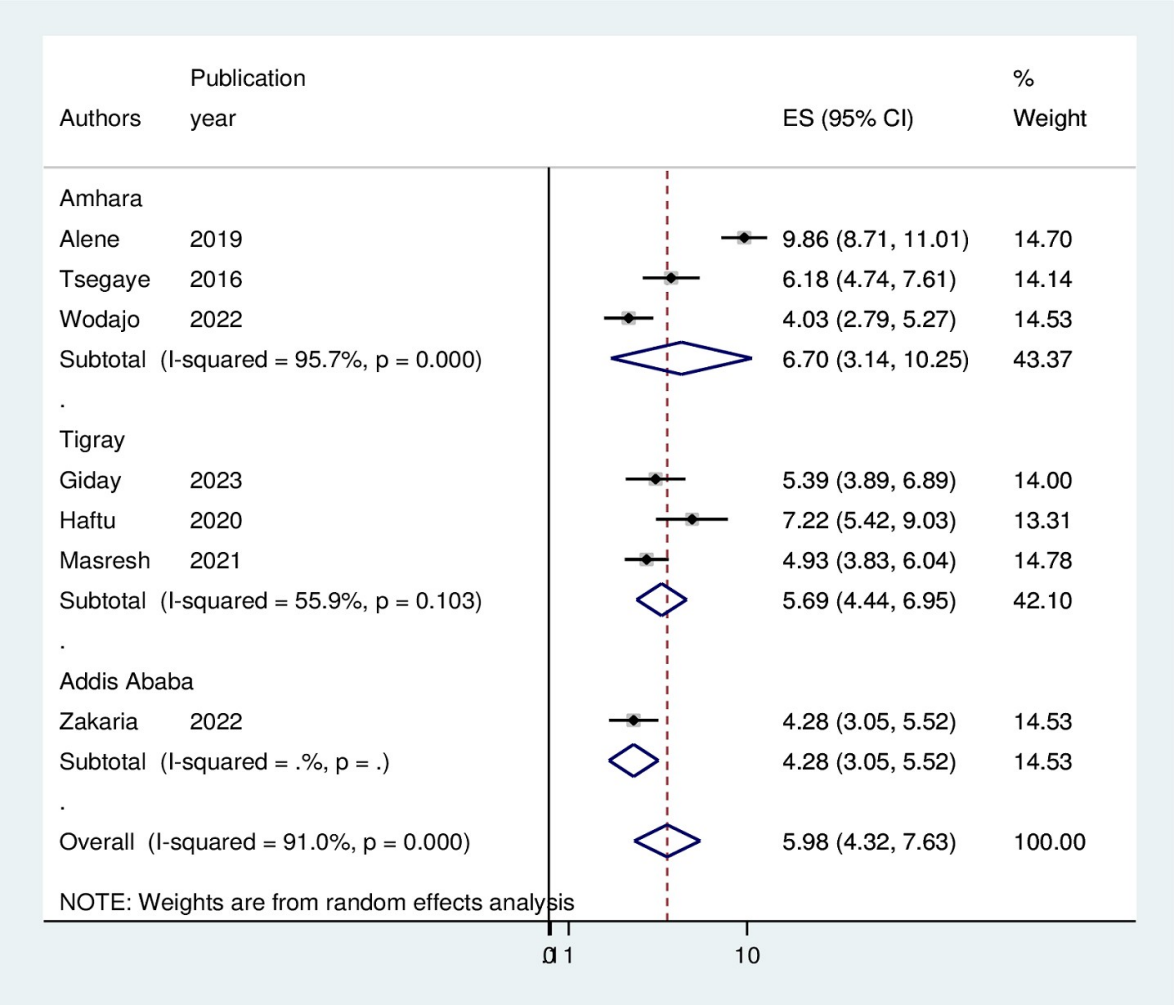
Forest plot displaying subgroup analysis on the pooled incidence of treatment failure among patients with HIV on second-line antiretroviral therapy in Ethiopia, 2023. **Abbreviations:** ES, effect Size; CI, Confidence Interval.

**Fig 4:**
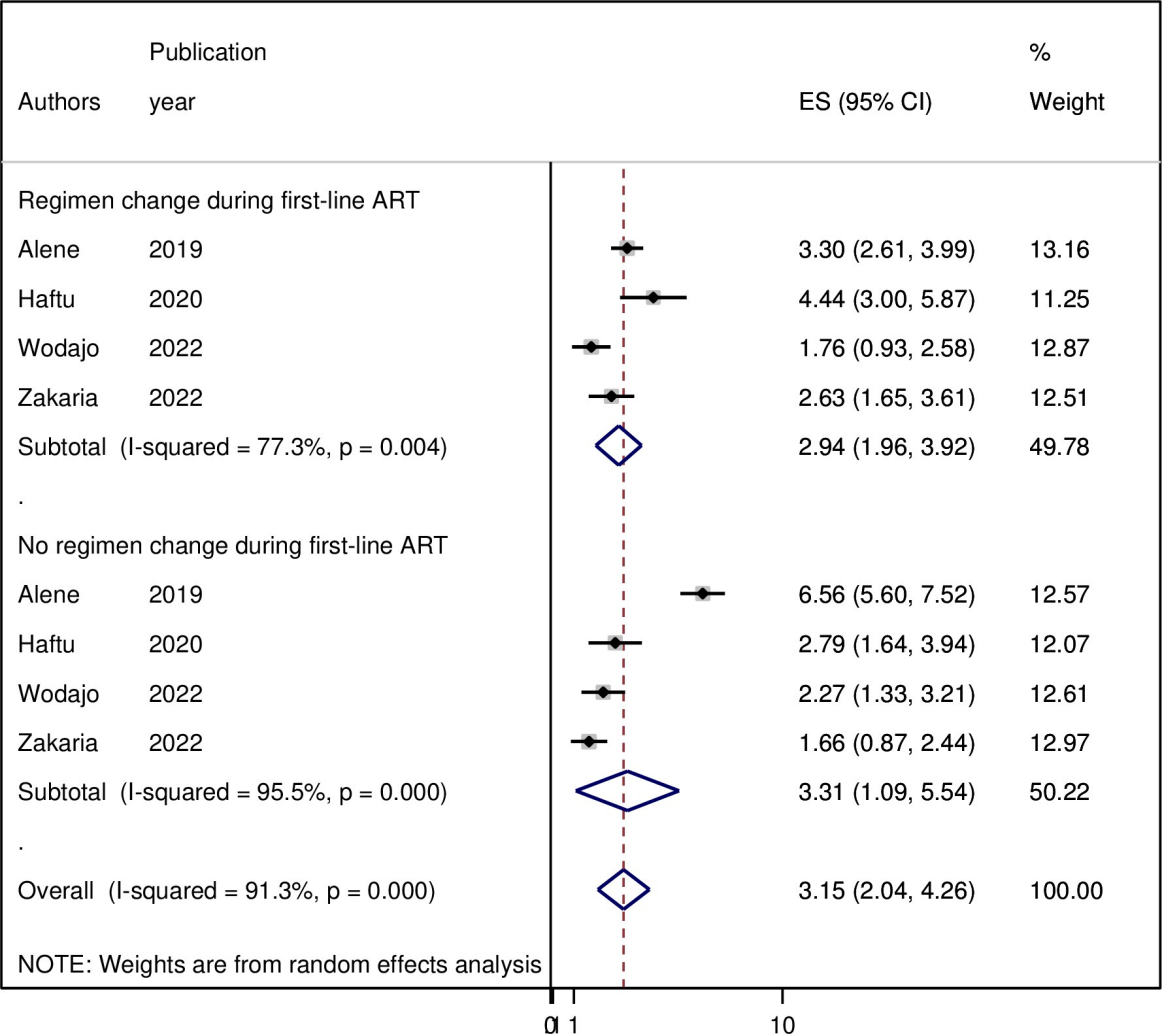
Forest plot displaying subgroup analysis by regimen changes on the pooled incidence of treatment failure among patients with HIV on second-line antiretroviral therapy in Ethiopia, 2023. **Abbreviations:** ES, effect Size; CI, Confidence Interval.

**Fig 5:**
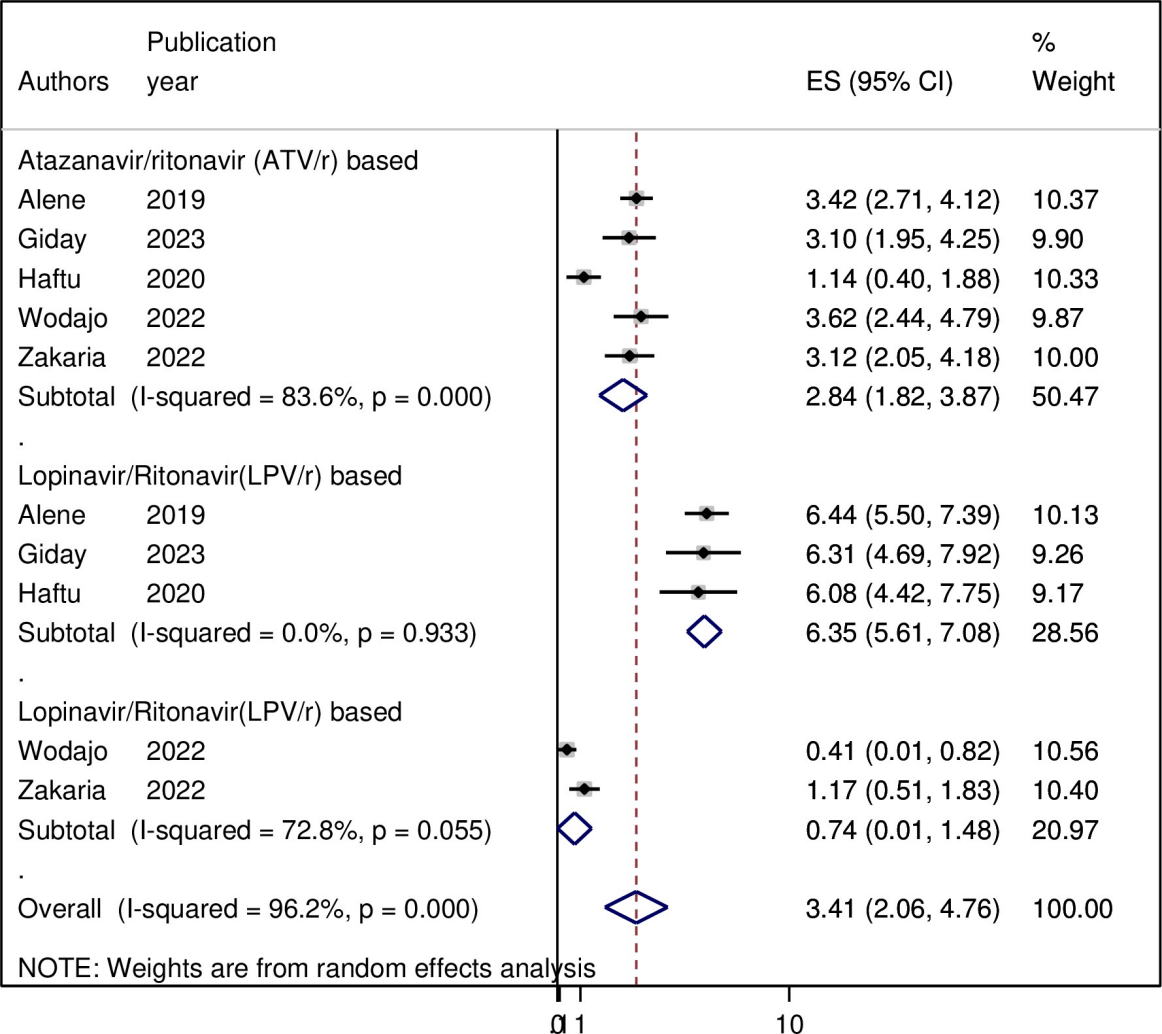
Forest plot displaying subgroup analysis by types of second line drugs on the pooled incidence of treatment failure among patients with HIV on second-line antiretroviral therapy in Ethiopia, 2023. **Abbreviations:** ES, effect Size; CI, Confidence Interval.

Because of the high heterogeneity observed across the studies (I2 = 90.1%, p < 0.001), subgroup analysis was performed based on the study area (regions where the studies were conducted). As per region, the highest pooled incidence of treatment failure was observed in Amhara region 6.7 (95% CI = 3.14, 10.25) per 100 person-years of observation and the lowest was in Tigray 5.69 (95% CI = 4.44, 6.95) per 100 person-years of observation. However, as shown in the figure the difference was not significant across the regions (Fig 3). Moreover, a sub-group analysis by types of second line ART regimen revealed that the incidence of second-line ART failure was 6.35 per 100 person-years of observation and 2.84 per 100 person-years of observation on ATV/r based and lPV/r based drugs developed second line ART failure, respectively (Fig 4). Sub-group analysis based on regimen change history during first-line ART indicated that the incidence of treatment failure was 3.31 per 100 person-years of observation among patients with HIV who had history of ART drug regimens (Fig 5).

### Publication bias

**Fig 6:**
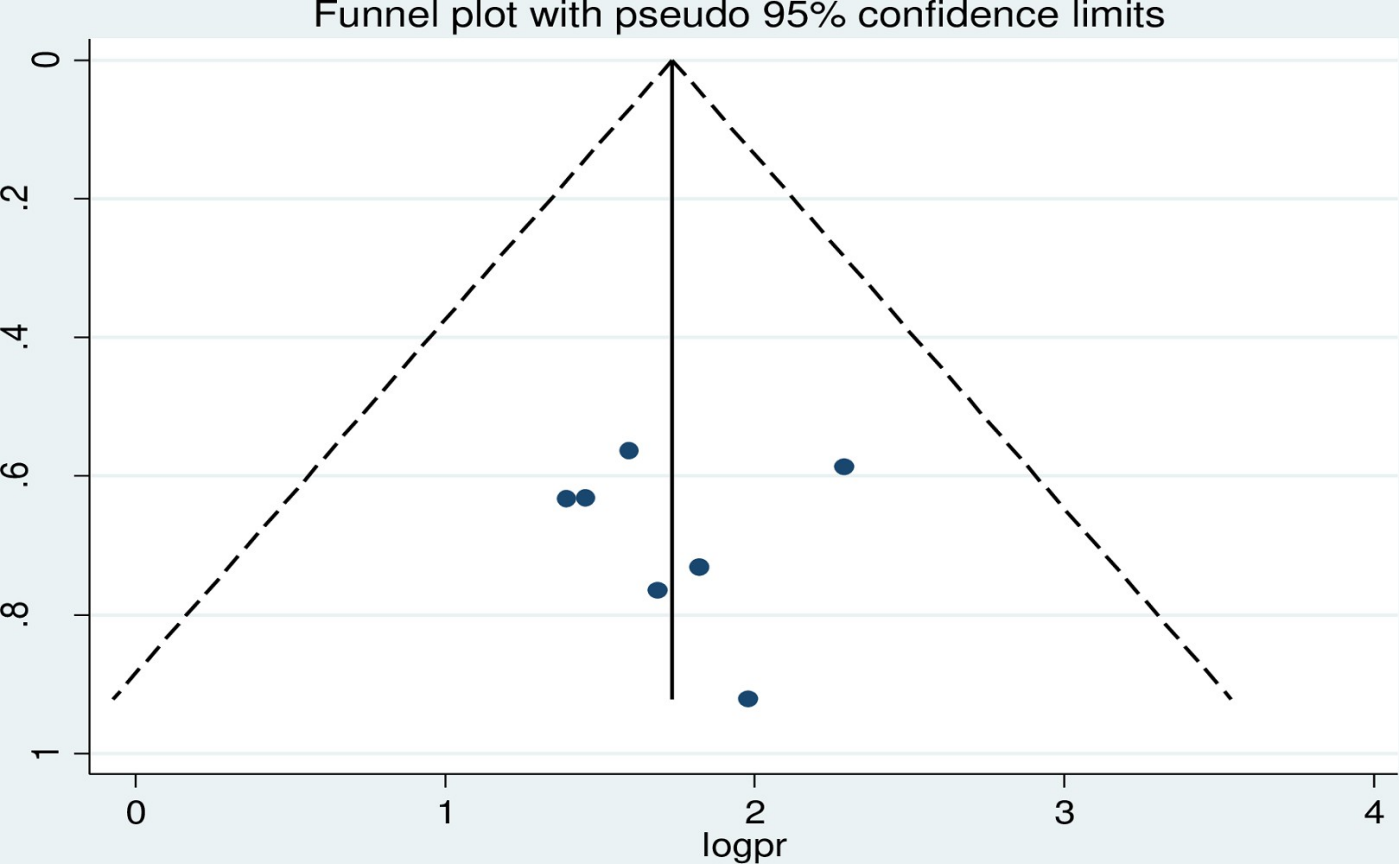
Forest plot displaying publication bias on the pooled incidence of treatment failure among patients with HIV on second-line antiretroviral therapy in Ethiopia, 2023.

To assess potential publication bias, funnel plots were visually inspected and statistically supported using Begg’s and Egger’s tests. A funnel plot showed symmetrical distribution of the included studies in a large inverted funnel, indicating no publication bias (Fig 6). This was supported by Begg’s and Egger’s tests, which revealed no publication bias among the studies included to estimate the pooled incidence of second-line ART failure among adults with HIV in Ethiopia, with p-values of (p <0.83).

### Sensitivity analysis

**Fig 7:**
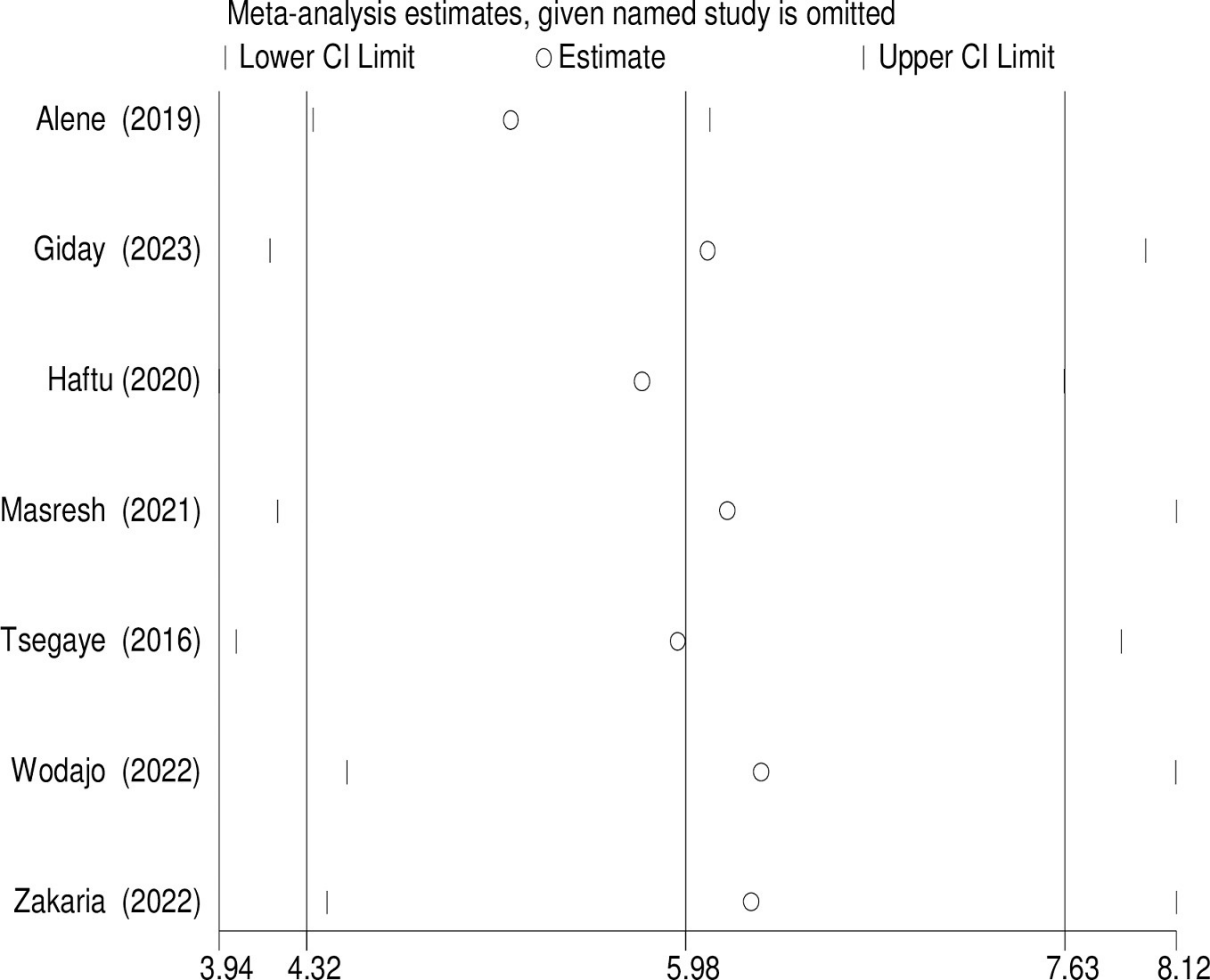
Forest plot displaying sensitivity analysis on the pooled incidence of treatment failure among patients with HIV on second-line antiretroviral therapy in Ethiopia, 2023.

Sensitivity analysis was used to assess the effect of a single study on second-line ART treatment failure among PLWHIV on second-line ART regimens in Ethiopia by excluding each study at a time. The findings revealed that no single study showed a statistically significant difference in the pooled incidence of second-line ART treatment failure among PLWHIV on second-line ART regimens in Ethiopia (Fig 7).

### Predictors of second-line antiretroviral treatment failure

To identify risk factors of second-line ART treatment failure, variables such as age, duration of follow-up, Advanced WHO clinical stage, ART drug adherence, baseline CD4 count, Body mass index and tuberculosis co-infection were extracted from the included studies. Finally, four variables were identified as independent predictors of second-line ART treatment failure: poor ART drug adherence, CD4 count < 100cells/mm^3^ at change, advanced WHO clinical stage and tuberculosis co-infection.

### Association between treatment failure and CD4 count at changing regimens

**Fig 8:**
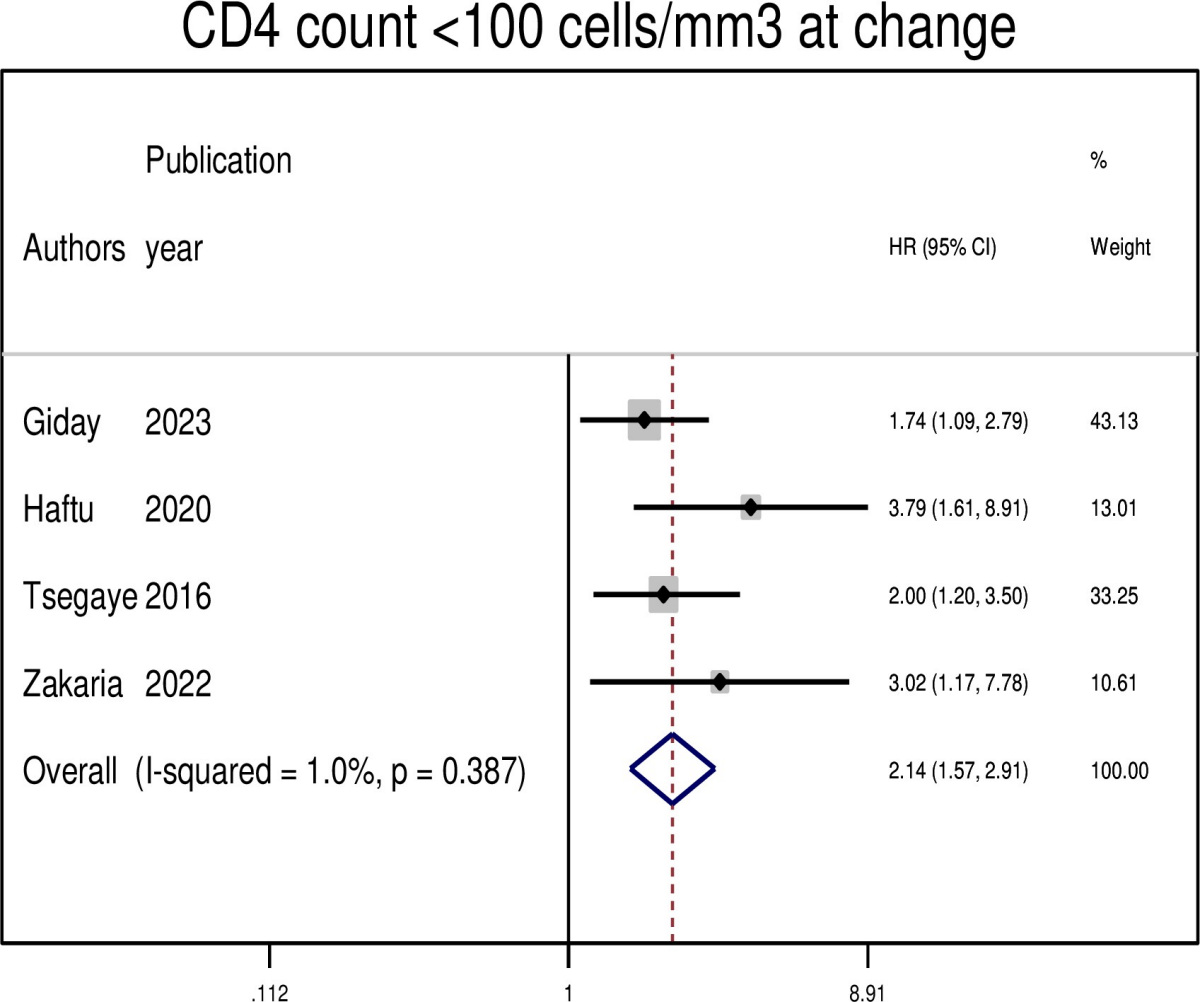
The pooled hazard ratio of the association between CD4 count and treatment failure among PLWHIV on second-line antiretroviral therapy in Ethiopia, 2023.

Four primary studies reported a low CD4 count of < 100cells/mm^3^ at switch as a risk factor for treatment failure among patients with HIV on second-line treatments. In this meta-analysis, the pooled hazard ratio revealed that patients with a CD4 count < 100cells/mm^3^ at switch were 2.14 times more likely to experience treatment failure (AHR: 2.14, 95% CI: 1.57, 2.91) (Fig 8).

#### Association between treatment failure and advanced WHO clinical stage at switch

**Fig 9:**
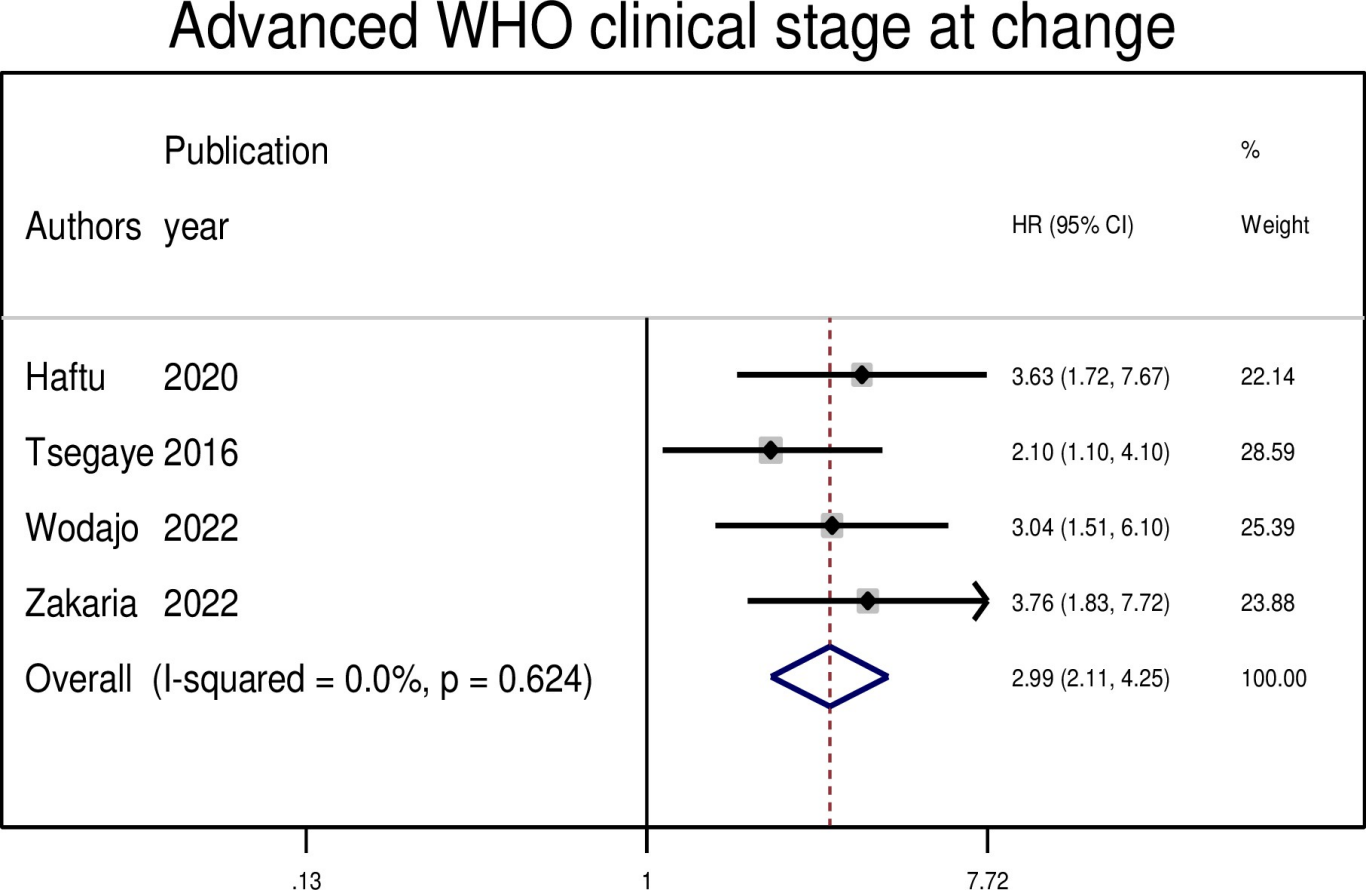
The pooled hazard ratio of the association between advanced WHO clinical stage and treatment failure among PLWHIV on second-line antiretroviral therapy in Ethiopia, 2023.

The WHO clinical stage at switch was found to be a risk factor for failure of second-line HIV therapies in four primary studies. The pooled risk of second-line treatment failure was almost three times higher among PLWHIV with advanced WHO clinical stage at switch than among those with WHO clinical stage I/II (AHR: 2.98, 95% CI: 2.11, 4.25) (Fig 9).

#### Association between treatment failure and poor ART drug adherence

**Fig 10:**
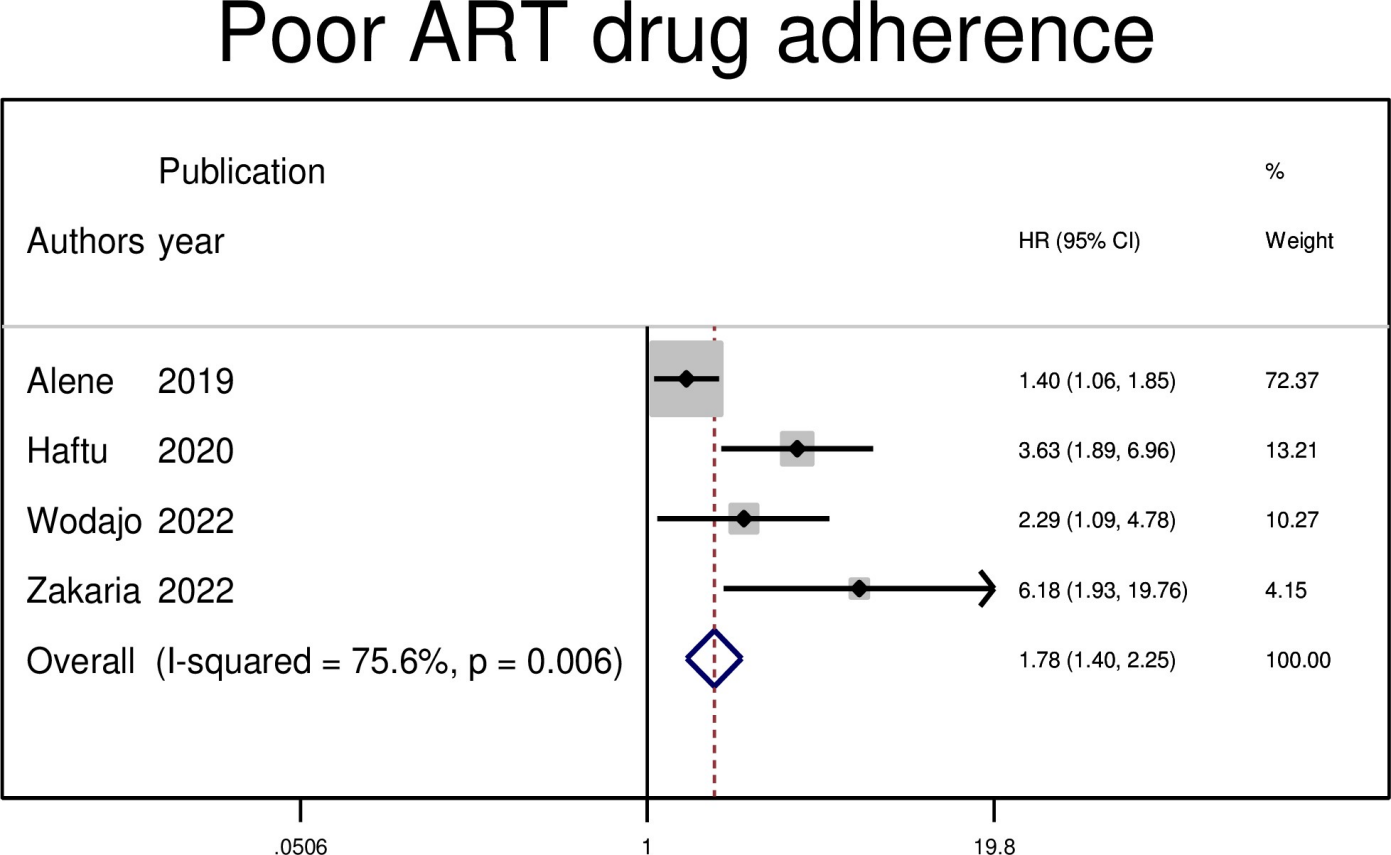
The pooled hazard ratio of the association between level of ART drug adherence and treatment failure among PLWHIV on second-line antiretroviral therapy in Ethiopia, 2023.

Among patients with HIV on second-line treatments, advanced WHO clinical stage at switch was associated with treatment failure in four primary studies. The hazard of treatment failure was nearly two times higher among those who had poor adherence to ART drugs compared to those who had good levels of adherence (AHR: 1.78, 95% CI: 1.4, 2.25) (Fig 10).

#### Association between treatment failure and tuberculosis co-infection

**Fig 11:**
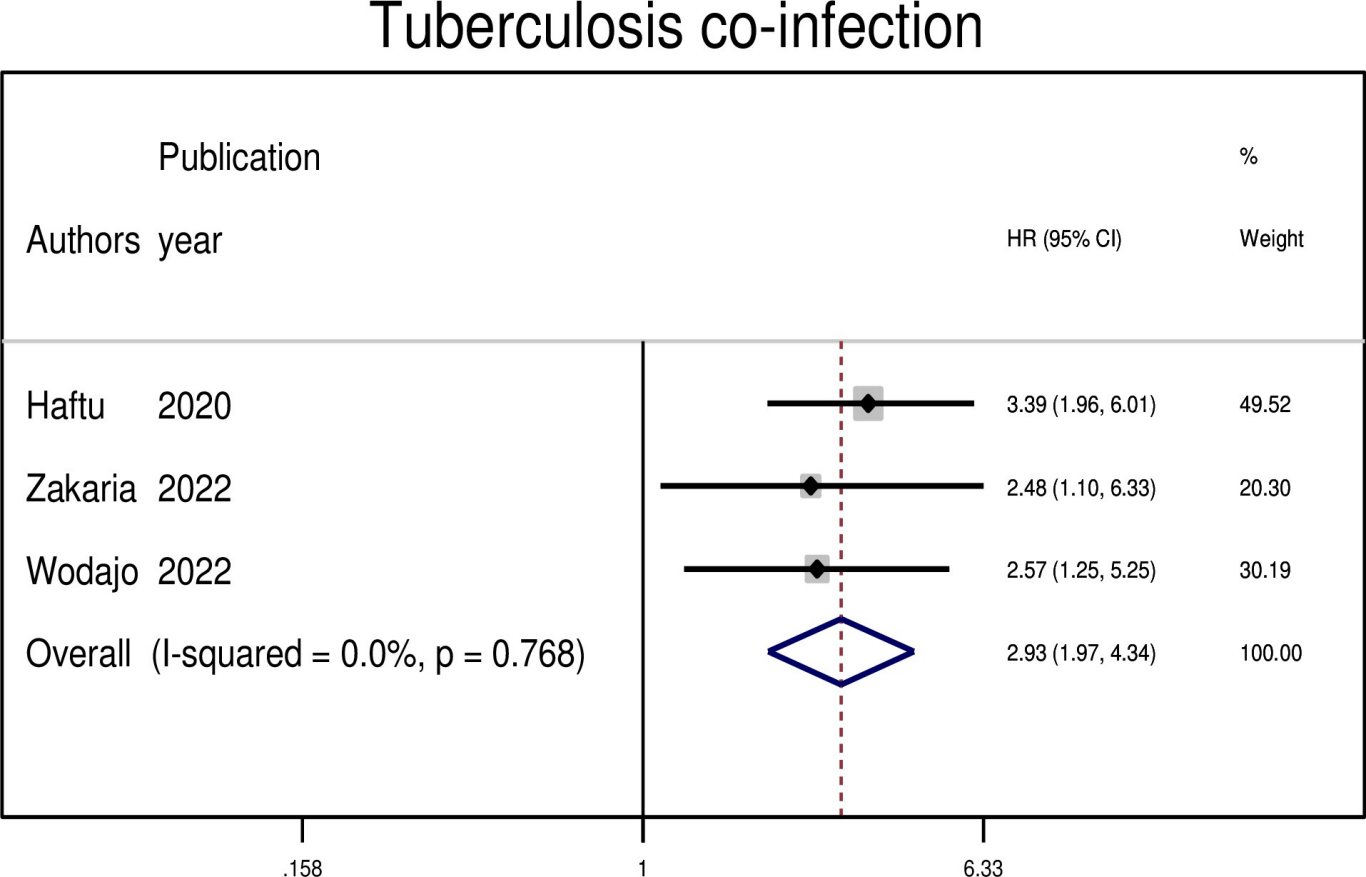
The pooled hazard ratio of the association between opportunistic infection and treatment failure among PLWHIV on second-line antiretroviral therapy in Ethiopia, 2023.

In three primary studies, having tuberculosis co-infection was associated with treatment failure among patients with HIV on second-line ART treatments. The hazard of treatment failure was 2.93 times higher among those who had tuberculosis co-infection as compared to those who had not (AHR: 2.93, 95% CI: 1.93, 4.34) (Fig 11).

## Discussion

Second-line ART treatment failure increases the risk of drug resistance, compromises the quality of life, and increases the need for expensive third-line treatment options. Therefore, determining the incidence and predictors of second-line treatment failure provides insights for designing contextually relevant strategies and policies. In this study, the incidence of second-line treatment failure was 5.98 (95% CI: 4.32, 7.63) per 100 person-years of observation. The incidence of viral failure in our study is similar to the UNAIDS goal of 5% of patients with undetectable viral load [25] indicating with continued efforts, Ethiopia can continue to make progress in the fight against HIV/AIDS and improve the health outcomes of its population. This finding was also nearly similar to that of studies conducted in a multicenter study in Asia 8.8 per 100 person-years) [26] and Myanmar 7.9 per 100 person-years) [27]. However, this finding was lower than that reported in a meta-analysis of sub-Saharan Africa (15.0%) [9] and India (15%) [28]. Variations in the study population and design may account for this disparity. For example, this review estimated the pooled incidence using person-years of follow-up as a denominator, whereas the studies in SSA estimated the prevalence, which could result in an overestimation of the incidence of treatment failure. In addition, this could be due to a complex interplay of factors related to healthcare systems, resources, disease patterns, patient demographics, and patient adherence to treatment. This study highlights the need for tailored approaches to address the challenges faced in providing effective HIV care in Ethiopia.

In the subgroup analysis, the likelihood of second-line antiretroviral treatment (ART) failure varies depending on the drugs used. The pooled incidence of second-line ART failure was 7.49 per 100 person-years of observation among those HIV patient on LPV/r based ART drug regimens and 6.72 per 100 person-years of observation among those HIV patient on ATV/r based ART drug regimens. Compared to ATV/r, LPV/r is associated with a higher risk of treatment failure due to factors such as drug potency, resistance profiles, and potential interactions with other medications [29]. Additionally, LPV/r may promote the development of drug-resistant HIV variants, which can result in virological failure in patients on LPV/r-based regimens. The numerous drug interactions associated with LPV/r can also affect drug levels, efficacy, and toxicity [30]. To prevent second-line ART treatment failure, it is important to address adherence issues, monitor drug interactions, and carefully consider the specific drug regimens used. Sustainable monitoring and individualized care are crucial for optimizing treatment outcomes.

In this study, the predictors of second-line ART failure were identified. Poor adherence to ART drugs, opportunistic infection, CD4 count < 100cells/mm^3^ at switch, and advanced WHO clinical stage III/IV at switching were identified as risk factors for second-line ART failure. Poor adherence to ART drugs has been identified as a risk factor for second-line antiretroviral treatment failure. The likelihood of second-line ART treatment failure was approximately three times higher among those with poor adherence to second-line ART drugs. This finding is consistent with those of studies conducted in South Africa, Vietnam, and other resource-limiting countries [31–33]. This might be because adherence to antiretroviral medication (ART) is critical to the therapeutic outcome of those living with HIV [34]. This is because people who do not closely follow their doctor’s prescription may not take their medications as directed. Moreover, the reason for this could be that, in the event of non-adherence, the blood concentration of antiretroviral drugs is insufficient to inhibit the replication of the viral RNA, which results in the failure of second-line antiretroviral therapy and the creation of drug-resistant viruses.

Advanced WHO clinical stage at the switch to second-line antiretroviral treatment is a risk factor for treatment failure. Similar findings were reported in studies in South Africa [35], Uganda [36], and India [37]. This could be explained by a variety of possible reasons, such as drug resistance to first-line medication during treatment, making second-line medication less effective [38]. In addition, delayed diagnosis and treatment might have already progressed to an advanced stage of HIV and may have a higher viral load and lower CD4 cell count, which reduces treatment effectiveness and increases the risk of treatment failure. Moreover, adherence to the complex medication regimen required for second-line treatment can lead to treatment failure [39]. Therefore, proper monitoring, support, and adherence education can help mitigate these risks and improve the treatment outcomes.

In this study, a CD4 count <100 cells/mm3 at the switch was significantly associated with a higher risk of treatment failure. The incidence of second-line treatment failure was found to be higher in patients with a CD4 count < 100 cells/mm3. This finding is consistent with previous studies conducted in Malawi [40], South Africa [33], and Vietnam [32]. A low CD4 count is often associated with a high probability of opportunistic infections, which may contribute to treatment failure.

Lastly, tuberculosis co-infection was also found to be a significant factor associated with treatment failure among patients on ART. Studies in South Africa and Uganda have shown that co-infection is a determining factor for ART treatment failure [41, 42]. This may be due to the presence of advanced opportunistic infections, which may deplete CD4 counts and compromise immunity, or the fact that medications for co-infection treatment and ART together can contribute to double burden side effects.

### Limitation of the study

Despite the study being nationally based, there were limitations such as only including articles published in English and only representing studies from three regions and one administrative town of the country, which may have biased the estimate of treatment failure incidence for the entire Ethiopian context. Lastly, some of the findings were discussed with primary studies because only a few national and international systematic reviews and meta-analyses were available. Therefore, it is essential to consider the potential impacts of these factors when interpreting reports.

## Conclusion

In conclusion, the research findings indicate the pooled incidence of second-line treatment failure was 5.89 per 100 person-years of observation. This implies that out of every 100 persons-year of follow-up approximately six patients experienced treatment failure. The study identified a higher risk of second line ART failure among patients who had poor level of ART drug adherence, advanced WHO clinical stage at switch, CD4 count <100 cells/mm^3^ at switch and presence of tuberculosis co-infection. Hence, strategies targeting the identified risk factors are essential for improving the effectiveness of second-line ART.

## Supporting information

S1 Checklist PRISMA 2020 checklist. (DOC)

S2 Checklist JBI standardized checklist. (DOCX)

S1 Table Risk of bias assessment tool. (DOCX)

## References

[1] Organization WH. Global update on HIV treatment 2013: results, impact and opportunities. 2013.

[2] Heath K, Levi J, Hill A. The Joint United Nations Programme on HIV/AIDS 95–95–95 targets: worldwide clinical and cost benefits of generic manufacture. AIDS. 2021;35(1):S197–S203. doi: 10.1097/QAD.0000000000002983 34115649

[3] Organization WH. Global health observatory (GHO) data: HIV/AIDS. Erişim adresi: https://www who int/gho/hiv/en/(Erişim Tarihi: 0112 19). 2018.

[4] EPHA. HIV Related Estimates and Projections for Ethiopia–2017. Addis Ababa. 2017.

[5] Organization WH. Consolidated guidelines on the use of antiretroviral drugs for treating and preventing HIV infection: recommendations for a public health approach: World Health Organization; 2016. 27466667

[6] Jiamsakul A, Azwa I, Zhang F, Yunihastuti E, Ditangco R, Kumarasamy N, et al. Treatment modification after second-line failure among people living with HIV in Asia-Pacific. Antiviral therapy. 2020;25(7):377–87. 33843656 10.3851/IMP3388 PMC8275121

[7] Frehiwot N, Mizan K, Seble M, Fethia K, Tekalign M, Zelalem T. National guidelines for comprehensive HIV prevention, care and treatment. Addis Ababa: Ministry of Health. 2014.

[8] Kwobah CM, Mwangi AW, Koech JK, Simiyu GN, Siika AM. Factors associated with first-line antiretroviral therapy failure amongst HIV-infected African patients: a case-control study. 2012.

[9] Edessa D, Sisay M, Asefa F. Second-line HIV treatment failure in sub-Saharan Africa: A systematic review and meta-analysis. PLoS One. 2019;14(7):e0220159. doi: 10.1371/journal.pone.0220159 31356613 PMC6663009

[10] Abdissa A, Yilma D, Fonager J, Audelin AM, Christensen LH, Olsen MF, et al. Drug resistance in HIV patients with virological failure or slow virological response to antiretroviral therapy in Ethiopia. BMC infectious diseases. 2014;14(1):1–7. doi: 10.1186/1471-2334-14-181 24708645 PMC4234735

[11] Alene M, Awoke T, Yenit MK, Tsegaye AT. Incidence and predictors of second-line antiretroviral treatment failure among adults living with HIV in Amhara region: a multi-centered retrospective follow-up study. BMC Infectious Diseases. 2019;19(1):1–9. 31288748 10.1186/s12879-019-4243-5 PMC6617674

[12] Wedajo S, Degu G, Deribew A, Ambaw F. Treatment failure, death, and predictors among PLWHIV on second-line antiretroviral therapy in Dessie Comprehensive Specialized Hospital, northeast Ethiopia: A retrospective cohort study. Plos one. 2022;17(6):e0269235. doi: 10.1371/journal.pone.0269235 35648771 PMC9159605

[13] Tsegaye AT, Wubshet M, Awoke T, Alene KA. Predictors of treatment failure on second-line antiretroviral therapy among adults in northwest Ethiopia: a multicentre retrospective follow-up study. BMJ open. 2016;6(12):e012537. doi: 10.1136/bmjopen-2016-012537 27932339 PMC5168604

[14] Sigaloff KC, Hamers RL, Wallis CL, Kityo C, Siwale M, Ive P, et al. Second-line antiretroviral treatment successfully resuppresses drug-resistant HIV-1 after first-line failure: prospective cohort in Sub-Saharan Africa. The Journal of infectious diseases. 2012;205(11):1739–44. doi: 10.1093/infdis/jis261 22448003

[15] Patrikar S, Shankar S, Kotwal A, Basannar D, Bhatti V, Verma R, et al. Predictors of first line antiretroviral therapy failure and burden of second line antiretroviral therapy. medical journal armed forces india. 2017;73(1):5–11. doi: 10.1016/j.mjafi.2016.09.008 28123238 PMC5221360

[16] Ramadhani HO, Bartlett JA, Thielman NM, Pence BW, Kimani SM, Maro VP, et al., editors. Association of first-line and second-line antiretroviral therapy adherence. Open forum infectious diseases; 2014: Oxford University Press. 10.1093/ofid/ofu079 PMC4281791 25734147

[17] Endalamaw A, Mekonnen M, Geremew D, Yehualashet FA, Tesera H, Habtewold TD. HIV/AIDS treatment failure and associated factors in Ethiopia: meta-analysis. BMC Public Health. 2020;20:1–12. 31959136 10.1186/s12889-020-8160-8 PMC6971997

[18] Ayele G, Tessema B, Amsalu A, Ferede G, Yismaw G. Prevalence and associated factors of treatment failure among HIV/AIDS patients on HAART attending University of Gondar Referral Hospital Northwest Ethiopia. BMC immunology. 2018;19:1–13. 30558580 10.1186/s12865-018-0278-4 PMC6296084

[19] Parums DV. Review articles, systematic reviews, meta-analysis, and the updated preferred reporting items for systematic reviews and meta-analyses (PRISMA) 2020 guidelines. Medical science monitor: international medical journal of experimental and clinical research. 2021;27:e934475–1. 34421116 10.12659/MSM.934475 PMC8394590

[20] Hoy D, Brooks P, Woolf A, Blyth F, March L, Bain C, et al. Assessing risk of bias in prevalence studies: modification of an existing tool and evidence of interrater agreement. Journal of clinical epidemiology. 2012;65(9):934–9. doi: 10.1016/j.jclinepi.2011.11.014 22742910

[21] Gidey K, Mache A, Hailu BY, Asgedom SW, Tassew SG, Nirayo YL. Second-Line Antiretroviral Treatment Outcomes and Predictors in Tigray Region, Ethiopia. Infection and Drug Resistance. 2023:4903–12. doi: 10.2147/IDR.S419348 37534062 PMC10390760

[22] Zakaria HF, Raru TB, Hassen FA, Ayana GM, Merga BT, Debele GR, et al. Incidence and Predictors of Virological Failure Among Adult HIV/AIDS Patients on Second-Line Anti-Retroviral Therapy, in Selected Public Hospital of Addis Ababa, Ethiopia: Retrospective Follow-Up Study. HIV/AIDS-Research and Palliative Care. 2022:319–29. doi: 10.2147/HIV.S367677 35836751 PMC9275424

[23] Zenebe Haftu A, Desta AA, Bezabih NM, Bayray Kahsay A, Kidane KM, Zewdie Y, et al. Incidence and factors associated with treatment failure among HIV infected adolescent and adult patients on second-line antiretroviral therapy in public hospitals of Northern Ethiopia: multicenter retrospective study. PloS one. 2020;15(9):e0239191. doi: 10.1371/journal.pone.0239191 32986756 PMC7521713

[24] Masresha SA, Kidie AA, Alen GD, Mulaw GF, Feleke FW, Kassaw MW, et al. Virological Failure and its Predictors among Human Immunodeficiency Virus Infected Individuals on Second Line Antiretroviral Treatment in North‐East Ethiopia, 2021. Pharmacoepidemiology and Drug Safety. 2023. doi: 10.1002/pds.5625 36974512

[25] Frescura L, Godfrey-Faussett P, Feizzadeh A A, El-Sadr W, Syarif O, Ghys PD, et al. Achieving the 95 95 95 targets for all: a pathway to ending AIDS. PLoS One. 2022;17(8):e0272405. doi: 10.1371/journal.pone.0272405 35925943 PMC9352102

[26] Boettiger DC, Nguyen VK, Durier N, Bui HV, Sim BLH, Azwa I, et al. Efficacy of second-line antiretroviral therapy among people living with HIV/AIDS in Asia: results from the TREAT Asia HIV observational database. JAIDS Journal of Acquired Immune Deficiency Syndromes. 2015;68(2):186–95. doi: 10.1097/QAI.0000000000000411 25590271 PMC4296907

[27] Kyaw NTT, Kumar AM, Oo MM, Oo HN, Kyaw KWY, Thiha S, et al. Long-term outcomes of second-line antiretroviral treatment in an adult and adolescent cohort in Myanmar. Global health action. 2017;10(1):1290916. doi: 10.1080/16549716.2017.1290916 28594295 PMC5496085

[28] Salvi S, Raichur P, Kadam D, Sangle S, Gupte N, Nevrekar N, et al. Virological failure among people living with HIV receiving second-line antiretroviral therapy in Pune, India. BMC infectious diseases. 2022;22(1):1–8. 36528762 10.1186/s12879-022-07894-2 PMC9758821

[29] Rahim M, Hassan Y, Fahrni ML. Predictor factors for treatment failure among patients on second line antiretroviral therapy. Age. 2014;24(3):0.001.

[30] Huang X, Xu L, Ding H, Ma P, Liu S, Chen X, et al. Six-year immunologic recovery and virological suppression of HIV patients on LPV/r-based second-line antiretroviral treatment: a multi-center real-world cohort study in China. Frontiers in pharmacology. 2019;10:454969. doi: 10.3389/fphar.2019.01455 31920648 PMC6917650

[31] Murphy RA, Sunpath H, Castilla C, Ebrahim S, Nguyen H, Kuritzkes DR, et al. Second-line antiretroviral therapy: long-term outcomes in South Africa. JAIDS Journal of Acquired Immune Deficiency Syndromes. 2012;61(2):158–63. doi: 10.1097/QAI.0b013e3182615ad1 22692090 PMC3767995

[32] Thao VP, Quang VM, Wolbers M, Anh ND, Shikuma C, Farrar J, et al. Second-line HIV therapy outcomes and determinants of mortality at the largest HIV referral center in Southern Vietnam. Medicine. 2015;94(43). doi: 10.1097/MD.0000000000001715 26512561 PMC4985375

[33] Pujades-Rodríguez M, Balkan S, Arnould L, Brinkhof MA, Calmy A. Treatment failure and mortality factors in patients receiving second-line HIV therapy in resource-limited countries. Jama. 2010;304(3):303–12. doi: 10.1001/jama.2010.980 20639564

[34] B Nachega J C Marconi V, U van Zyl G, M Gardner E, Preiser W, Y Hong S, et al. HIV treatment adherence, drug resistance, virologic failure: evolving concepts. Infectious Disorders-Drug Targets (Formerly Current Drug Targets-Infectious Disorders). 2011;11(2):167–74. 10.2174/187152611795589663 PMC5072419 21406048

[35] Fox MP, Van Cutsem G, Giddy J, Maskew M, Keiser O, Prozesky H, et al. Rates and predictors of failure of first-line antiretroviral therapy and switch to second-line ART in South Africa. JAIDS Journal of Acquired Immune Deficiency Syndromes. 2012;60(4):428–37. doi: 10.1097/QAI.0b013e3182557785 22433846 PMC3392418

[36] Kazooba P, Mayanja BN, Levin J, Masiira B, Kaleebu P. Virological failure on first-line antiretroviral therapy; associated factors and a pragmatic approach for switching to second line therapy–evidence from a prospective cohort study in rural South-Western Uganda, 2004–2011. Pan African Medical Journal. 2018;29(1):1–16. 30061969 10.11604/pamj.2018.29.191.11940 PMC6061817

[37] Chakravarty J, Sundar S, Chourasia A, Singh PN, Kurle S, Tripathy SP, et al. Outcome of patients on second line antiretroviral therapy under programmatic condition in India. BMC infectious diseases. 2015;15:1–11. 26572102 10.1186/s12879-015-1270-8 PMC4647630

[38] Langford SE, Ananworanich J, Cooper DA. Predictors of disease progression in HIV infection: a review. AIDS research and therapy. 2007;4:1–14. 17502001 10.1186/1742-6405-4-11 PMC1887539

[39] Vidya Vijayan K, Karthigeyan KP, Tripathi SP, Hanna LE. Pathophysiology of CD4+ T-cell depletion in HIV-1 and HIV-2 infections. Frontiers in immunology. 2017;8:580. doi: 10.3389/fimmu.2017.00580 28588579 PMC5440548

[40] Court R, Leisegang R, Stewart A, Sunpath H, Murphy R, Winternheimer P, et al. Short term adherence tool predicts failure on second line protease inhibitor-based antiretroviral therapy: an observational cohort study. BMC Infectious Diseases. 2014;14:1–7. 25472544 10.1186/s12879-014-0664-3 PMC4266950

[41] Eshun-Wilson I, Taljaard JJ, Nachega JB. Sub-optimal CD4 T-lymphocyte responses among HIV infected patients who develop TB during the first year of ART. Journal of AIDS & clinical research. 2012;3(135). doi: 10.4172/2155-6113.1000135 22442755 PMC3308912

[42] Hermans SM, Kiragga AN, Schaefer P, Kambugu A, Hoepelman AI, Manabe YC. Incident tuberculosis during antiretroviral therapy contributes to suboptimal immune reconstitution in a large urban HIV clinic in sub-Saharan Africa. PloS one. 2010;5(5):e10527. doi: 10.1371/journal.pone.0010527 20479873 PMC2866328

