# Supplementary material for: Second-line anti-retroviral treatment failure and its predictors among patients with HIV in Ethiopia: A systematic review and meta-analysis
Source: PLOS Glob Public Health. 2024 Apr 23;4(4):e0003138. doi: 10.1371/journal.pgph.0003138 (PMC11037545; doi:10.1371/journal.pgph.0003138)
Supplement: S2 Checklist — (DOCX) [file pgph.0003138.s002.docx]

Supplemental file 2: Quality assessment for the included Studies to Second-Line Anti-retroviral Treatment Failure and Its Predictors among HIV Patients in Ethiopia using JBI standardized checklist

| Authors | Appropriateness of the source population list | Describe study setting and participant | Valid and reliable exposure measurement | Objective and standard criteria for measurement | Identified confounder | Strategies to deal with confounders | Valid and reliable outcome measurement | Appropriate statically analysis | Number of ‘yes’ |
| --- | --- | --- | --- | --- | --- | --- | --- | --- | --- |
| Alene et al (2019) | Yes | Yes | No | Yes | Yes | Yes | Yes | Yes | 7/8=87.5 |
| Giday et al (2023) | Yes | Yes | Yes | Yes | No | No | Yes | Yes | 6/8=75 |
| Haftu et al (2020) | Yes | Yes | No | Yes | Yes | No | Yes | Yes | 6/8=75 |
| Masresh et al (2021) | Yes | Yes | No | Yes | Yes | Yes | Yes | Yes | 7/8=87.5 |
| Tsegaye et al (2016) | Yes | Yes | No | Yes | Yes | Yes | Yes | Yes | 6/8=87.5 |
| Wodajo et al (2022) | Yes | Yes | Yes | Yes | No | No | Yes | Yes | 7/8=75 |
| Zakaria et al (2022) | Yes | Yes | Yes | Yes | No | No | Yes | Yes | 6/8=75 |
