# Supplementary material for: Second-line anti-retroviral treatment failure and its predictors among patients with HIV in Ethiopia: A systematic review and meta-analysis
Source: PLOS Glob Public Health. 2024 Apr 23;4(4):e0003138. doi: 10.1371/journal.pgph.0003138 (PMC11037545; doi:10.1371/journal.pgph.0003138)
Supplement: S1 Table — (DOCX) [file pgph.0003138.s003.docx]

S1 Table: Assessment of risk of bias for the included studies

| Item | External validity | | | | Internal validity | | | | | |  | |
| --- | --- | --- | --- | --- | --- | --- | --- | --- | --- | --- | --- | --- |
|  | Representativeness s of the target population | Representativeness s of the sampling frame | Radom sampling g or census | Minimal response e bias | Data were collected d directly | Acceptable e case definition used in the study | Valid and reliable measurement t | The same mode of data collection n for all study subject | Appropriate e length of prevalence period for parameter of interest | Appropriate numerators and denominator s of interest | No of yes | Summary of risk of bias |
| Alene et al (2019)(11) | Yes | Yes | No | Yes | Yes | No | Yes | Yes | Yes | Yes | 8 | Low-  risk |
| Giday et al (2023) (19) | Yes | Yes | Yes | Yes | No | No | Yes | Yes | Yes | Yes | 8 | Low risk |
| Haftu et al (2020)(21) | Yes | Yes | No | Yes | Yes | No | Yes | Yes | Yes | Yes | 8 | Low- risk |
| Masresha et al (2021)(22) | Yes | Yes | No | Yes | Yes | Yes | Yes | Yes | Yes | Yes | 9 | Low – risk |
| Tsegaye et al (2016)(13) | Yes | Yes | No | Yes | Yes | Yes | Yes | Yes | Yes | Yes | 9 | Low- risk |
| Wodajo et al (2022) | Yes | Yes | Yes | Yes | Yes | No | Yes | Yes | Yes | Yes | 9 | Low- risk |
| Zakaria et al (2022)(20) | Yes | Yes | No | Yes | Yes | Yes | Yes | Yes | Yes | Yes | 9 | Low- risk |
